# Exploring the Use of Computed Tomography Enterography in the Evaluation of Small Bowel Disease: A Prospective Study

**DOI:** 10.7759/cureus.60915

**Published:** 2024-05-23

**Authors:** Dhanya S B, Sunil H C, Gowthami G S, Ravi Kumar Yeli, Suhas C N, Praveen Kumar M

**Affiliations:** 1 Department of Radiology, Jagadguru Sri Shivarathreeshwara (JSS) Medical College, Mysuru, IND; 2 Department of Radiology, Kanachur Institute of Medical Sciences, Mangalore, IND; 3 Department of Pediatrics, Al-Ameen Medical College and Research Center (RC), Vijayapur, IND; 4 Department of Radiology, Bijapur Lingayat District Educational (BLDE) (Deemed to be University) Shri B. M. Patil Medical College and Research Centre, Vijayapur, IND; 5 Department of Radiology, Krishna Scans, Vellore, IND; 6 Department of Radiology, Sri Jayadeva Institute of Cardiovascular Sciences and Research, Mysuru, IND

**Keywords:** noninvasive, inflammation, mannitol, imaging, small bowel disorders, ct enterography

## Abstract

Background

A major development in noninvasive imaging modalities, computed tomographic enterography (CTE) has a number of benefits over conventional computed tomography (CT) and capsule endoscopy. Through the utilization of multidetector computed tomography (MDCT) technology, CTE expedites the assessment of small bowel diseases, especially in those segments that are not accessible through traditional endoscopy. This study's main goal is to thoroughly evaluate CTE's diagnostic accuracy for a range of small intestinal conditions.

Methodology

In this investigation, which is a prospective observational study, 40 patients, 25 men and 15 women, with suspected small intestinal disorders and ages ranging from 10 to 70 underwent CTE. To evaluate diagnosis accuracy, a combination of clinical symptoms, imaging data, and histopathological/ultrasonography findings were evaluated. Throughout the research procedure, ethical issues and statistical analysis were incorporated to guarantee validity and adherence to ethical norms.

Results

The most frequent findings on CTE were bowel thickening and mucosal hyperenhancement, which were seen in 25 (62.5%) and 20 (50%) of the patients, respectively. The majority of patients (65%) exhibited both the ileal and jejunal loops to be adequately distended in grade III. In 35% of the patients, grade II distensibility of the ileal and jejunal loops was seen.

Conclusion

When it comes to accurately detecting small intestinal disorders, CTE is superior. It evaluates extraintestinal, mural, and intraluminal diseases with efficacy, particularly in places that are difficult to reach. It is essential for directing clinical decisions because of its capacity to assess disease activity prior to endoscopy and see consequences.

## Introduction

The small bowel section of the alimentary canal presents the greatest examination challenge because of its length, caliber, and overlapping loops [1]. Because of their caliber and size, the tiny intestinal loops are always under-evaluated by both conventional radiography and endoscopic modalities. The evaluation of small bowel disease's extra-enteric symptoms has been greatly enhanced by computed tomography (CT). On the other hand, it can only illustrate gut wall and luminal diseases [2].

With today's sophisticated multidetector-row computed tomography (MDCT) equipment, a novel and reliable method for characterizing small bowel illness is computed tomography enterography (CTE). This technique offers a thorough assessment and interpretation of the gut's luminal and mural characteristics, and it is utilized in CTE. Precisely representing the peri-enteric tissues enhances and broadens the evaluation of illness consequences [2,3]. This modality makes better three-dimensional reconstructions (volume rendering, surface shading, and multiplanar reconstruction display) and volume rendering via virtual CT endoscopic navigation possible [4]. The aforementioned technology in new scanners facilitates isotropic image collection in a single breath-hold and enables multiphasic high-resolution examination of intestinal characteristics [2,5].

Using narrow collimated slices and a large volume of contrast agent to highlight the small intestine wall and lumen properly, CTE varies from traditional abdominopelvic CT. In contrast to the conventional follow-through examination, this method evaluates small intestinal characteristics, such as hypervascular lesions and hyperenhancing segments, using an intravenous contrast agent and a neutral contrast material, such as water [6]. Water greatly influences the top part of the gastrointestinal (GIT) and is frequently utilized as a neutral contrast element. Due to its quick absorption, its application for the distal small bowel is constrained in clinical practice [7,8].

By increasing the osmolarity of the solution, an addition might result in sluggish water absorption [1]. One such addition is mannitol, an accessible and affordable neutral oral contrast agent. Mannitol's isoosmotic characteristic aids in sufficient small bowel distention, which makes CTE a useful modality for evaluating small bowel disorders [9]. To make CTE more accessible in decision-making for further care, the primary goal of this research was to study its function in identifying and characterizing small bowel disorders by employing mannitol for ideal intestinal distension.

## Materials and methods

Study design and setting

The current investigation was carried out at the Shri B. M. Patil Medical College Hospital and Research Centre in Vijayapur, in the department of radiodiagnosis. Forty-five patients who were hospitalized and sent at the Department of Radiodiagnosis for CTE because they may have small intestinal illnesses based on imaging, laboratory, or clinical findings participated in the prospective observational research. All patients who met the selection criteria and were between the ages of 10 and 70 years were included in the research. Consent was obtained or waived by all participants in this study. The Institutional Ethical Committee of Shri B. M. Patil Medical College Hospital and Research Centre, Vijayapur, issued approval IEC/Ref/No-142/17.

Selection Criteria

Inclusion criteria for the study encompassed patients exhibiting clinical suspicion of small bowel pathology, characterized by symptoms such as abdominal pain, features indicative of small bowel obstruction, vomiting, and chronic diarrhea. Exclusion criteria were established to ensure the safety and integrity of the study. Patients who declined to provide consent were pregnant, had documented iodinated contrast allergies, were unable to consume large volumes of fluid due to debilitation, possessed ileostomies from previous surgeries, or had comorbid conditions where fluid overload posed risks (such as cardiac or renal diseases) were excluded. Additionally, hemodynamically unstable patients with gastrointestinal bleeding and those with deranged blood urea and serum creatinine levels were ineligible. Patient histories, including age, sex, and any family history of similar diseases, were meticulously recorded, and eligible patients received thorough counseling regarding the study's nature and purpose. Relevant investigations were conducted to further assess patient eligibility and inform study outcomes.

Data sources and variables

The examination technique for CTE involved meticulous preparation and administration of oral contrast, where 20% of 450 ml of mannitol was mixed with 1350 ml of water to create 1800 ml of isoosmotic mannitol. Patients consumed the entire volume under supervision, with 500 ml ingested every 20 minutes over an hour, along with a 300 ml dose before scanning. Oral contrast intake and any associated problems were recorded.

CTE was conducted with a standardized protocol, scanning from the diaphragm to the pubic symphysis, utilizing parameters such as 120 kVp, 251 mAs, and a detector collimation of 64 x 0.625. Intravenous nonionic contrast media (350 mg/ml Omnipaque) was administered, with imaging during the enteric phase. Images were reviewed at dedicated workstations, and multiplanar and maximum intensity projection (MIP) reconstructions were performed. Post-acquisition, patients were observed for four hours to note any diarrhea or adverse effects from oral contrast, with side effects recorded. Follow-up interviews were conducted telephonically two days post-CTE.

Image recording and evaluation assessed the distension of the entire small bowel, graded as grade III, grade II, or grade I, and overall small bowel evaluation categorized as good, fair, or bad. Lesions, strictures, and abdominal organ details were also evaluated.

Statistical analysis

Frequency and percentage were employed as statistical measures to characterize the qualitative data gathered in the study. In addition to descriptive statistics, the diagnostic accuracy of MDC imaging in detecting abnormalities within the small intestine was rigorously evaluated to aid in the precise identification and diagnosis of patient lesions.

## Results

Of 40 patients, males were 25 (62.5 %) and females were 15 (37.5 %). The feasibility of CTE was evaluated based on the complete visibility of various intestinal segments via adequate distension. Table 1 illustrates the age distribution among subjects. The distribution of age ranges within the study population, consisting of 40 subjects, provides insights into the demographic composition. No individuals fell within the 0-10 age group. However, the 10-20 age bracket accounted for five subjects (12.5%), while the 20-30 age range comprised seven individuals (17.5%). A slightly higher representation was observed in the 30-40 age category, with nine participants (22.5%). The 40-50 age range exhibited the highest frequency, with 11 subjects (27.5%). Additionally, four individuals (10%) were in the 50-60 age group, while the 60-70 and 70+ age groups each included two subjects (5%).

**Table 1:** Age distribution among subjects

Age range	No. of subjects (%)
0-10	0 (0%)
10-20	5 (12.5%)
20-30	7 (17.5%)
30-40	9 (22.5%)
40-50	11 (27.5%)
50-60	4 (10%)
60-70	2 (5%)

Table 2 presents the CTE findings among the study population. Among the patients, bowel thickening was a prominent feature in 25 cases (62.5%), indicating potential pathological changes within the bowel wall. Conversely, 15 patients (37.5%) did not exhibit any significant thickening. Evaluation of bowel enhancement revealed notable findings, with 22 patients (55%) showing enhanced bowel segments suggestive of active inflammation or vascular changes, while 18 patients (45%) exhibited unenhanced bowel segments. Mucosal hyperenhancement, a characteristic sign of mucosal inflammation or irritation, was evident in 20 patients (50%), mirroring an equal proportion of patients lacking this feature. Mural stratification, a hallmark of deeper inflammatory changes within the bowel wall, was identified in 18 patients (45%), contrasting with 22 patients (55%) who did not display this characteristic stratified pattern.

**Table 2:** CTE findings among the study population CTE: Computed tomography enterography

CTE findings	Sample population (N = 40)	
	Present (%)	Absent (%)
Bowel thickening	25 (62.5%)	15 (37.5%)
Bowel enhancement	22 (55%)	18 (45%)
Mucosal hyperenhancement	20 (50%)	20 (50%)
Mural stratification	18 (45%)	22 (55%)

Table 3 outlines the spectrum of small bowel diseases diagnosed on CTE. Among the identified diagnoses, adhesive band pathology was observed in two cases (5%), while Crohn's disease presented in seven individuals (17.5%). Ileocecal (IC) tuberculosis exhibited the highest frequency, with eight patients (20%) diagnosed. Adenocarcinoma and ulcerative colitis were identified in three (7.5%) and four(10%) cases, respectively. Celiac disease and ischemic stricture each accounted for two cases (5%), while jejunitis, duodenitis, and ileitis collectively represented six cases (15%). Additionally, five subjects (12.5%) displayed normal small bowel findings.

**Table 3:** Spectrum of small bowel diseases diagnosed on CTE CTE: Computed tomography enterography

CTE diagnosis	Frequency	Percentage
Adhesive band	2	5%
Crohn’s disease	7	17.5%
Ileocecal tuberculosis	8	20%
Adenocarcinoma	3	7.5%
Ulcerative colitis	4	10%
Celiac disease	2	5%
Ischemic stricture	3	7.5%
Jejunitis/duodenitis/ileitis	6	15%
Normal	5	12.5%
Total	40	100%

## Discussion

Small bowel disease is a difficult public health issue of significant relevance and size in India that can be impacted by a broad range of illnesses. Bowel involvement can take many different forms, such as ischemic strictures, radiation enteritis, Crohn's disease, and iliotibial band (ITB). In the current study, the most prevalent complaint was abdominal discomfort, which was reported by 40 (100%) of the patients. Anorexia was reported by 25 (62.5%) of the cases. About five patients (12.5%) had a history of prior surgery, and 16 patients (40%) had minor intestinal obstruction upon presentation.

Bowel thickness and mucosal hyperenhancement were the most frequently seen CTE results in the current investigation, occurring in 25 (62.5 %) and 20 (50 %) patients, respectively. Results are in line with earlier research [10]. It was determined that intestinal diseases might affect the jejunum, ileum, or other colonic segments separately or in various combinations. For the purposes of our investigation, the IC junction was regarded as a component of the terminal ileum and the cecum as a component of the large intestine. The most frequently affected section in our research (47.73%) was the ileum, which is consistent with findings from prior investigations [10,11].

Mural wall thickness greater than 3 mm was referred to as mural thickening. Both nonneoplastic reasons, such as ulcerative colitis, radiation enteritis, ITB, Crohn's disease, ischemia strictures, and others, may result in mural thickness. Adenocarcinoma, gastrointestinal stromal tumor (GIST), metastases, and lymphoma are among the neoplastic reasons of thickening [12]. In 25 (62.5%) of the patients in the current investigation, the gut wall exhibited increased thickness. With three cases (7.5%) of neoplastic adenocarcinoma, nonneoplastic causes accounted for the majority of mural thickening (91.3%). Our results align with those of earlier researches [10-12].

Mural thickening was categorized as severe, >10 mm (Crohn's disease and neoplasms including lymphoma); moderate, 5-9 mm (Crohn’s disease, early adenocarcinoma, and lymphoma); and mild, 3-4 mm (infectious enteritis, mild Crohn’s disease). In the current investigation, among the 25 individuals exhibiting intestinal wall thickening, 19 (76 %) had mild-to-moderate thickening, and six (24 %) had severe thickening. This was consistent with a research by Megally et al. [13], wherein 81.8% of the patients in the nonneoplastic group had mild-to-moderate thickening. In the research by Megally et al. [13], 13 of the 17 patients (76.5%) exhibited substantial intestinal wall thickening, whereas both malignant cases (100%) showed marked bowel wall thickening.

While symmetrical and regular mural thickening is often found in benign diseases but can also be detected in neoplastic pathologies, asymmetric and irregular bowel wall thickening typically suggests malignant pathology [14]. Five (20%) of the patients in our research had asymmetrical thickening, whereas 20 (80%) of the cases had symmetrical thickening. In our investigation, asymmetrical thickening was seen in three Crohn's disease patients and in all 100 neoplastic cases. These results aligned with those of previous research [13].

In the current investigation, individuals with ulcerative colitis who had the colon diffusely affected by the disease were the two instances with the diffuse pattern of bowel involvement. Both of the bowel adenocarcinoma patients had focal involvement in our investigation. Focal thickening was brought on by both adenocarcinoma and TB in a different investigation conducted by Chamail et al. [15]. In the majority of cases (54%), localized thickening was linked to malignancy. The bulk (54%) of the thickening resulting from the tubercular etiology was segmental.

The majority of patients (65%) in the current research had acceptable grade III distension of their ileal and jejunal loops. In 35% of the patients, grade II distensibility of the ileal and jejunal loops was seen. Using mannitol as the contrast agent, 56% of the patients in a different research by Elamparidhi et al. [16] had grade II distensibility, while 40% had grade III distensibility. The participants in the present research tolerated CTE nicely. The most frequent side effect observed in eight (20%) instances was nausea. Of the patients, five (12.5%) reported experiencing post-procedural pain, and five complained of abdominal discomfort. Following the investigation, three patients (7.5%) reported experiencing diarrhea. Since none of the participants had vomiting, there were no further negative experiences. These results align with those of previous research [16-18].

The majority of individuals with jejunal wall thickening (>3mm) have been diagnosed with jejunal bowel disorders. Using Pearson chi-square, it can be shown that there is a statistically significant link (p < 0.001) between bowel disorders identified on CTE and jejunal wall thickening. The current investigation demonstrates that ileal wall thickening (>3mm) was present in most patients with CT diagnosis of bowel disorders, and the ileum was the location of presumed wall thickening. Using Pearson chi-square, it can be shown that there is a statistically significant link (p < 0.001) between ileal wall thickness and bowel disorders identified on CTE. These results are consistent with previous research by Yousuf et al. [19] in 2021 and Sahu et al. [20] in 2022, which found that CTE is more useful than other methods for diagnosing small intestinal disorders.

Limitations

Though this study had commendable outcomes, it had its share of limitations. One of the limitations was the small sample size, which can be expanded in future studies for a better understanding of the illness. Moreover, this study was carried out at a single center. Hence, geographical and different regional habits could have impacted the results, and therefore, larger studies spanning multiple organizations can be planned to take the field forward.

## Conclusions

With remarkable sensitivity and specificity, CTE is a very successful and well-tolerated diagnostic tool for small intestinal illnesses. CTE is an essential first diagnostic technique that is particularly useful for evaluating intraluminal, mural, and extraintestinal diseases, especially in difficult-to-reach sections. Its critical importance in maximizing diagnostic accuracy and directing clinical decision-making for small bowel disorders is highlighted by its capacity to assess disease activity prior to endoscopy and improve visualization of additional enteric problems.
